# Supplementary material for: New insights in photodynamic inactivation of Leishmania amazonensis: A focus on lipidomics and resistance
Source: PLoS One. 2023 Sep 15;18(9):e0289492. doi: 10.1371/journal.pone.0289492 (PMC10503701; doi:10.1371/journal.pone.0289492)
Supplement: S1 File — (DOCX) [file pone.0289492.s001.docx]

## Supporting Information


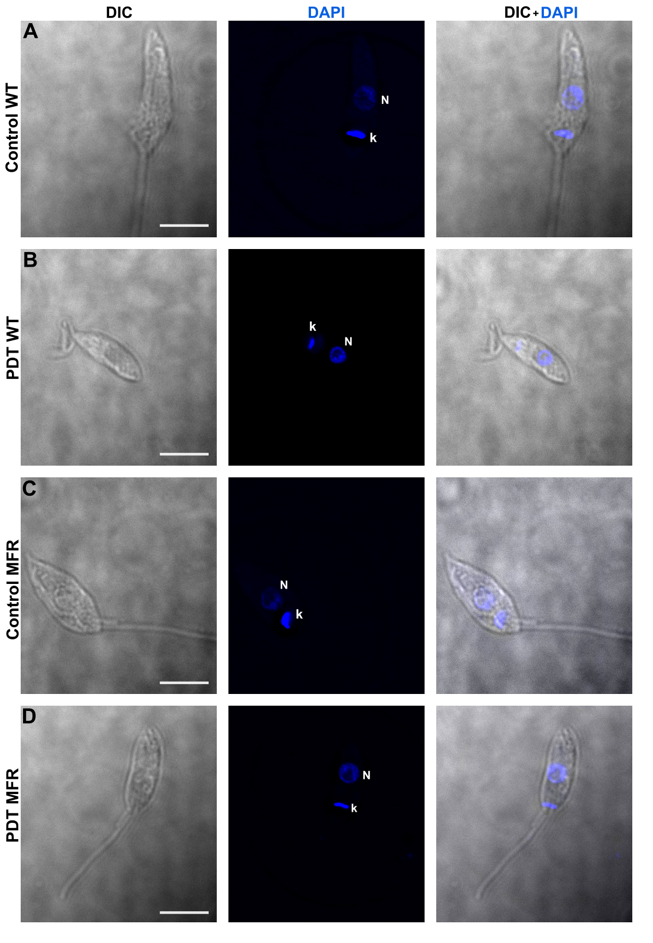


Figure S1 Differential interference contrast (DIC) and immunofluorescence staining images of WT and MFR *L. amazonensis* treated with PDT at 8 J/cm^2^ in the presence of 750 nM of DMMB. Nuclei and kinetoplast were stained with DAPI (blue fluorescence) directly after PDT. N = Nuclei; k = Kinetoplast. Bar = 5 µm.


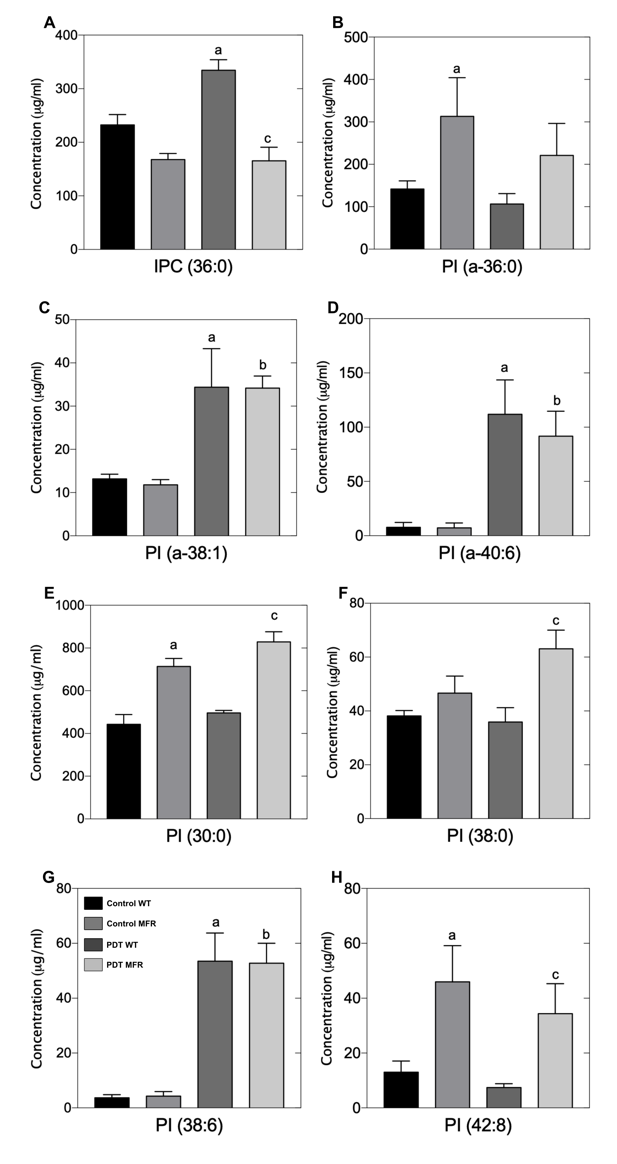


Figure S2. Inositol-containing molecular species of WT and MFR *L. amazonensis* promastigotes treated with PDT at 8 J/cm^2^ in the presence of 750 nM of DMMB. Precursor ion scanning for *m/z* 241 detected [M - H]^-^ PI and IPC ions from parasites total lipid extracts. A-H represents the concentrations of each individual molecular species of the corresponding untreated and treated WT and MFR phenotypes. “a” denotes statistically significant differences of PLs species compared to Control WT. “b” denotes statistically significant differences of PLs species compared to Control MFR. “c” denotes statistically significant differences of PLs species between PDT WT and PDT MFR.


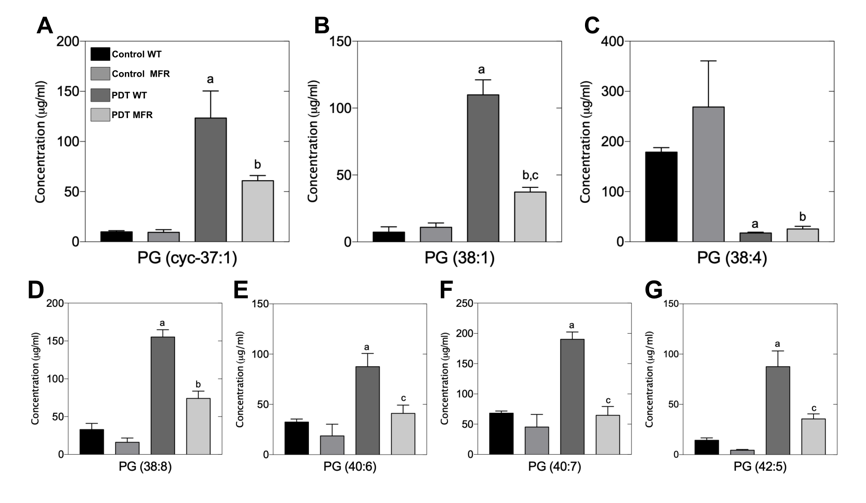


Figure S3. Phosphatidic acid (PA) and phosphatidylglycerol (PG) molecular species of WT and MFR *L. amazonensis* promastigotes treated with PDT at 8 J/cm^2^ in the presence of 750 nM of DMMB. Precursor ion scanning for *m/z* 153 [M-H]^-^ detected glycerophospholipids ions from parasites total lipid extracts.

A-G represents the concentrations of each individual molecular species of the corresponding untreated and treated WT and MFR phenotypes. “a” denotes statistically significant differences of PLs species compared to Control WT. “b” denotes statistically significant differences of PLs species compared to Control MFR. “c” denotes statistically significant differences of PLs species between PDT WT and PDT MFR.


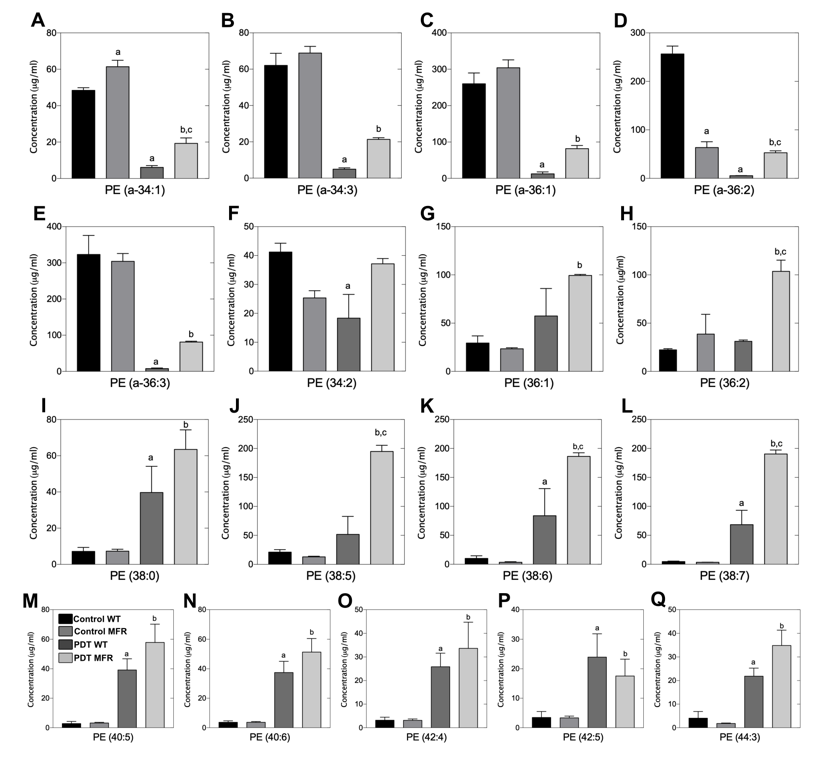


**Figure S4.** Phosphatidylethanolamine (PE) molecular species of WT and MFR *L. amazonensis* promastigotes treated with PDT at 8 J/cm^2^ in the presence of 750 nM of DMMB. Precursor ion scanning for *m/z* 196 detected [M - H]^-^ PE ions from parasites total lipid extracts. A-Q represents the concentrations of each individual molecular species of the corresponding untreated and treated WT and MFR phenotypes. “a” denotes statistically significant differences of PLs species compared to Control WT. “b” denotes statistically significant differences of PLs species compared to Control MFR. “c” denotes statistically significant differences of PLs species between PDT WT and PDT MFR.


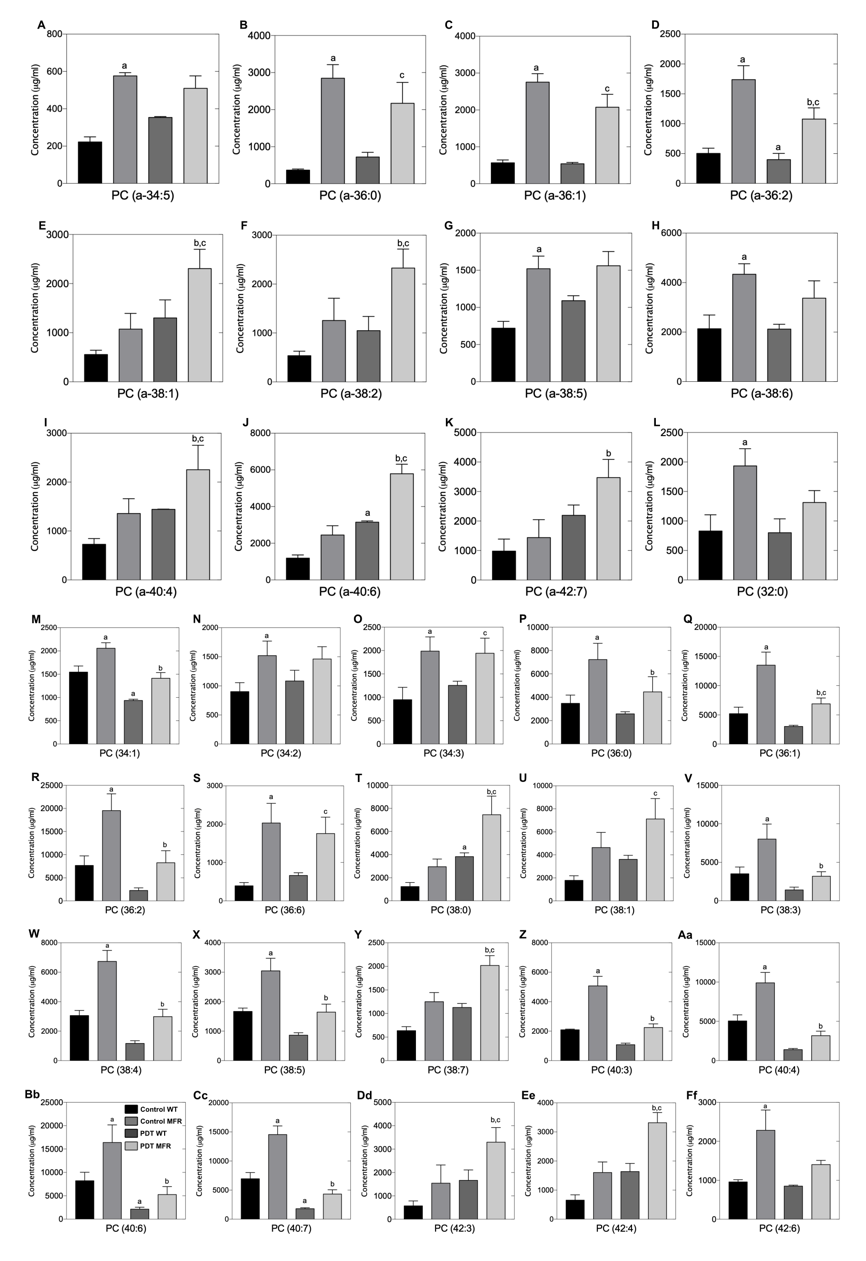


Figure S5. Phosphatidylcholine (PC) molecular species of WT and MFR *L. amazonensis* promastigotes treated with PDT at 8 J/cm^2^ in the presence of 750 nM of DMMB. Precursor ion scanning for *m/z* 184 [M + H]^+^ detected PI and IPC ions from parasite total lipid extracts. A-Ff represents the concentrations of each individual molecular species of the corresponding untreated and treated WT and MFR phenotypes. “a” Denotes statistically significant differences of PLs species compared to Control WT. “b” denotes statistically significant differences of PLs species compared to Control MFR. “c” denotes statistically significant differences of PLs species between PDT WT and PDT MFR.


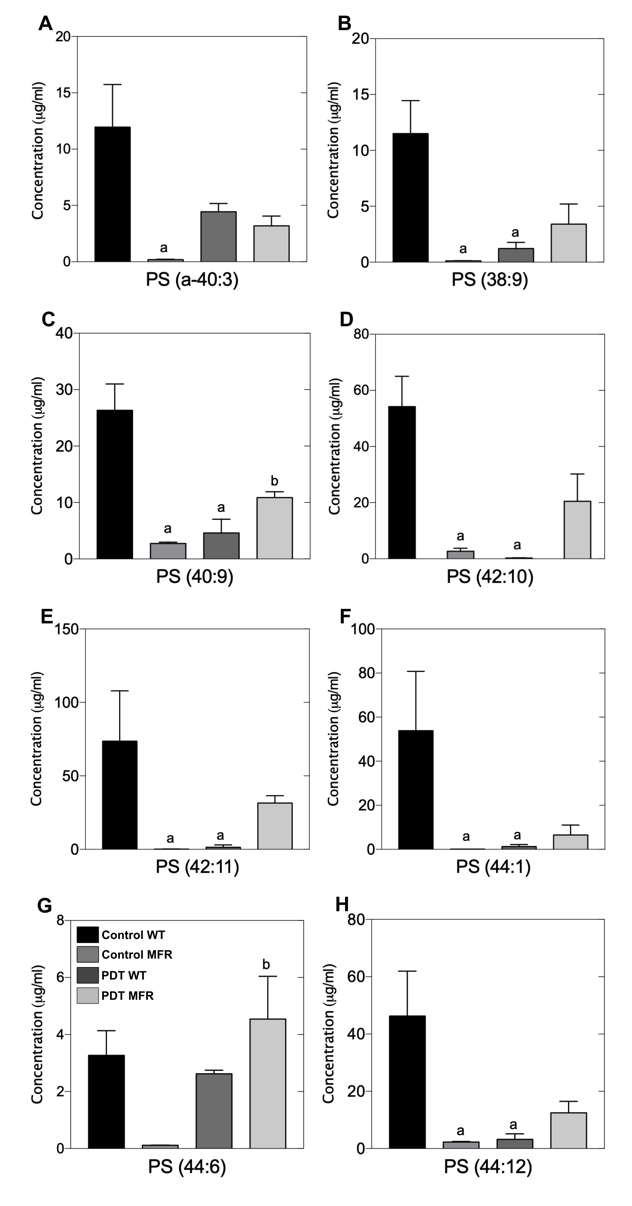


Figure S6. Phosphatidylserine (PS) molecular species of WT and MFR *L. amazonensis* promastigotes treated with PDT at 8 J/cm^2^ in the presence of 750 nM of DMMB. Neutral loss scanning for *m/z* 87 detected [M - H]^-^ PS ions from parasites total lipid extracts. A-H represents the concentrations of each individual molecular species of the corresponding untreated and treated WT and MFR phenotypes. “a” Denotes statistically significant differences of PLs species compared to Control WT. “b” denotes statistically significant differences of PLs species compared to Control MFR. “c” denotes statistically significant differences of PLs species between PDT WT and PDT MFR.

**
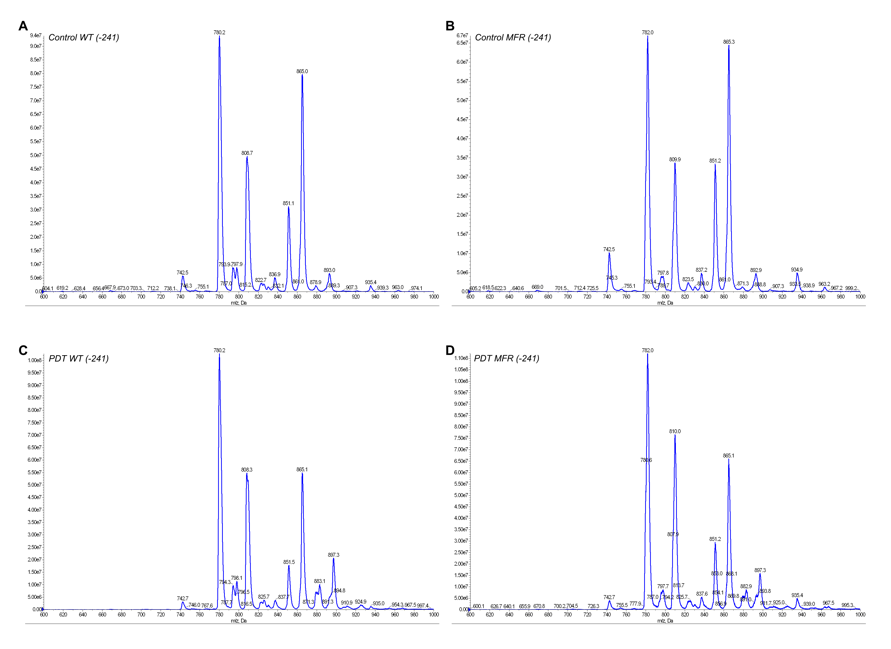
**

**Figure S7.** ESI-MS-MS negative ion spectra of inositol-containing PLs in WT and MFR *L. amazonensis* promastigotes. Precursor ion scanning for *m/z* 241 detected [M - H]^-^ PI and IPC ions from parasites total lipid extracts. WT (Wild-type); MFR (miltefosine-resistant).

**
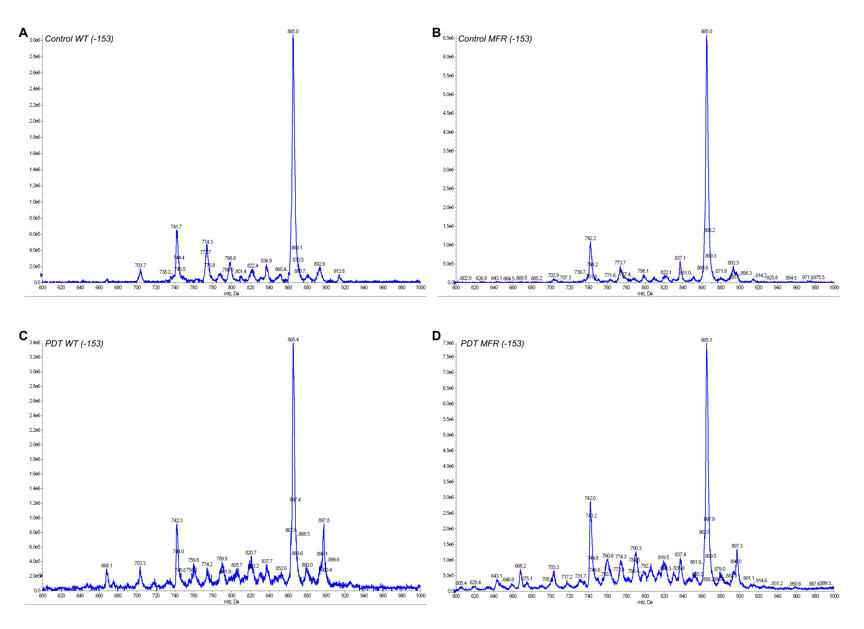
**

Figure S8. ESI-MS-MS negative ion spectra of PA and PG in in WT and MFR *L. amazonensis* promastigotes. Precursor ion scanning for *m/z* 153 detected [M - H]^-^ PA and PG ions from parasites total lipid extracts. WT (Wild-type); MFR (miltefosine-resistant).

**
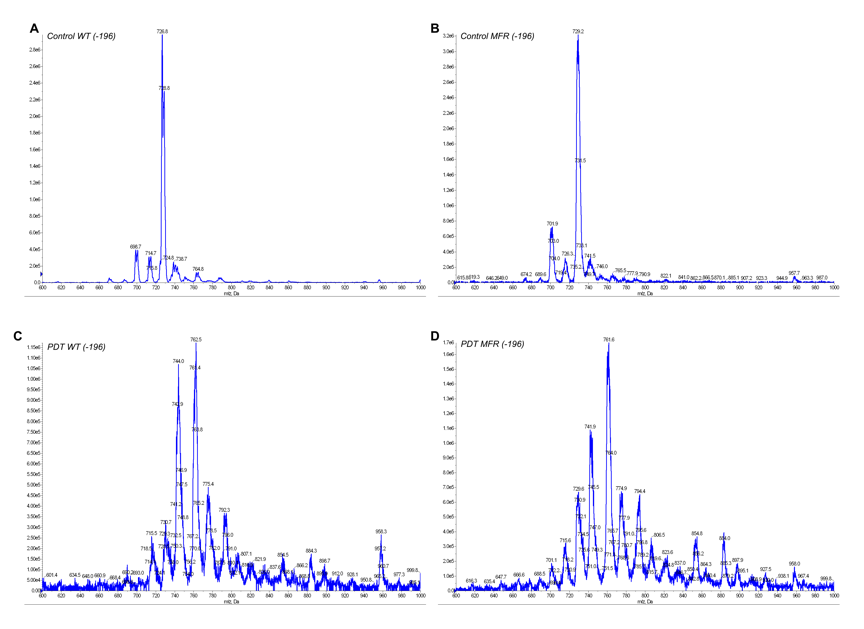
**

Figure S9. ESI-MS-MS negative ion spectra of PE in WT and MFR *L. amazonensis* promastigotes. Precursor ion scanning for *m/z* 196 detected [M - H]^-^ PE ions from parasites total lipid extracts. WT (Wild-type); MFR (miltefosine-resistant).

**
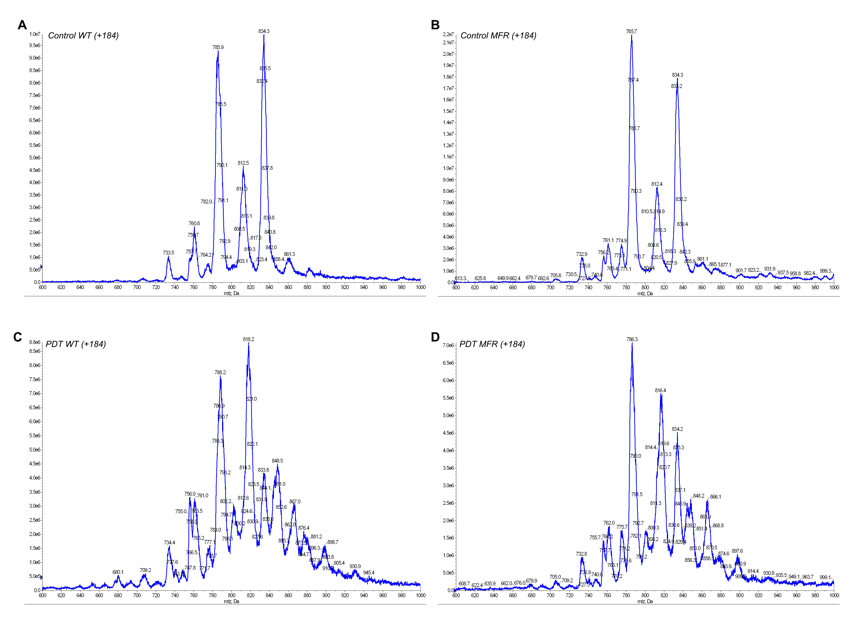
**

Figure S10. ESI-MS-MS positive ion spectra of PC in WT and MFR *L. amazonensis* promastigotes. Precursor ion scanning for *m/z* 184 detected [M + H]^+^ PC ions from parasites total lipid extracts. WT (Wild-type); MFR (miltefosine-resistant).

**
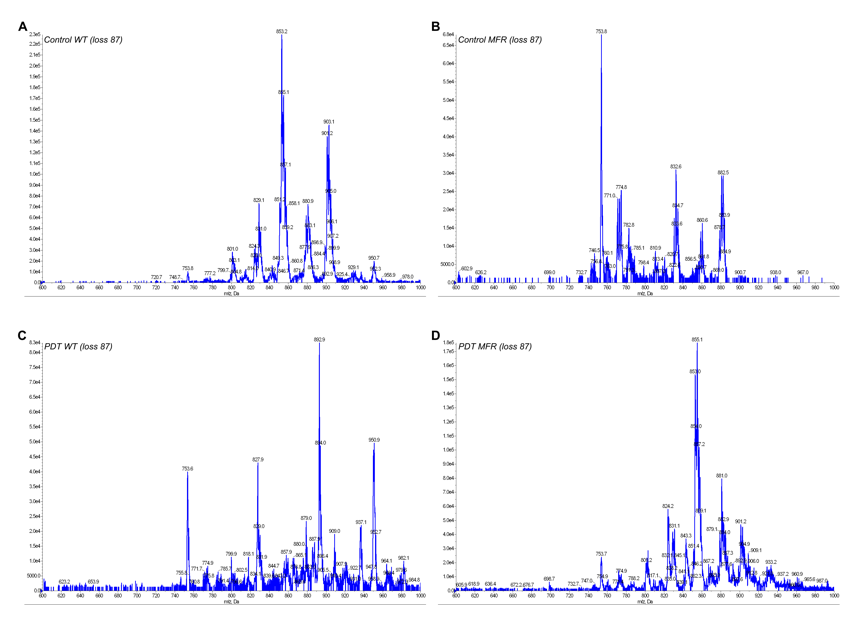
**

Figure S11. ESI-MS-MS negative ion spectra of PS in WT and MFR *L. amazonensis* promastigotes. Neutral loss scanning for *m/z* 87 detected [M - H]^-^ PS ions from parasites total lipid extracts. WT (Wild-type); MFR (miltefosine-resistant).

**Supplementary Table 1**: Mass spectrometric analysis of phospholipid species in *L. amazonensis*

**INOSITOLPHOSPHOCERAMIDE (IPC)**

| *m/z^a^* | Lipid component^b^ |
| --- | --- |
| 778.4  780.2  806.6  808.7 | IPC 34:1  IPC 34:0  IPC 36:1  IPC 36:0 |

**PHOSPHATIDYLINOSITOL (PI)**

| *m/z^a^* | Lipid component^b^ |
| --- | --- |
| 782.0  810.9 | 30:0  32:0 |
| 793.9  822.7  836.9  851.1  865.0  878.9  883.0  893.0  897.5  935.4 | a-32:2  a-34:1  34:0  a-36:0  36:0  a-38:1  38:6  38:0  a-40:6  42:8 |

**PHOSPHATIDIC ACID (PA)**

| *m/z^a^* | Lipid component^b^ |
| --- | --- |
| 668.2  703.7  741.7 | 34:4  36:1  a-40:2 |

**PHOSPHATIDYLGLYCEROL (PG)**

| *m/z^a^* | Lipid component^b^ |
| --- | --- |
| 742.4  759.4  774.3  789.9  798.8  805.7  821.7  822.4  853.0 | 34:4  cyc-37:1  36:2  38:8  38:4  38:1  40:7  40:6  42:5 |

**PHOSPHATIDYLETHANOLAMINE SPECIES (PE)**

| *m/z^a^* | Lipid component^b^ |
| --- | --- |
| 698.7  700.6  701.6  714.7  715.8  726.8  728.8  729.1  731.2  738.7  740.6  742.5  744.5  760.2  762.3  764.8  775.0  792.1  794.4  796.3  798.3  822.1  823.1  854.1 | a-34:3  a-34:2  a-34:1  34:2  34:1  a-36:3  a-36:2  a-36:1  a-36:0  36:4  36:3  36:2  36:1  38:7  38:6  38:5  38:0  40:6  40:5  40:4  40:3  42:5  42:4  44:3 |

**PHOSPHATIDYLCHOLINE SPECIES (PC)**

| *m/z^a^* | Lipid component ^b^ |
| --- | --- |
| 734.5  738.3  756.2  758.7  760.7  762.8  772.4  774.5  776.6  777.5  785.9  788.5  790.1  792.1  794.4  800.1  802.0  803.1  808.5  811.0  812.5  816.1  818.5  820.5  823.4  832.4  834.4  835.5  837.8  839.8  842.0  848.5  856.4  858.4  861.3  866.4  867.2 | 32:0  a-34:5  34:3  34:2  34:1  34:0  a-36:2  a-36:1  a-36:0  36:6  36:2  36:1  36:0  a-38:6  a-38:5  a-38:2  a-38:1  38:7  38:5  38:4  38:3  38:1  38:0  a-40:6  a-40:4  40:7  40:6  40:5  40:4  40:3  40:2  a-42:7  a-42:3  a-42:1  42:6  42:4  42:3 |

**PHOSPHATIDYLSERINE (PS)**

| *m/z^a^* | Lipid component^b^ |
| --- | --- |
| 743.5  753.8 | a-34:2  34:5 |
| 801.0 | 38:9 |
| 828.0 | a-40:3 |
| 829.1 | 40:9 |
| 853.2 | 42:11 |
| 855.4 | 42:10 |
| 880.9 | 44:12 |
| 892.5 | 44:6 |
| 903.1 | 44:1 |

1. Observed [M+H]^+^ or [M-H]^-^ ions, mass over charge from survey scans as described in material methods.
2. Peak identities refer to total number of carbon atoms and double bonds.

a =(alkylacyl)
