# Supplementary material for: New insights in photodynamic inactivation of Leishmania amazonensis: A focus on lipidomics and resistance
Source: PLoS One. 2023 Sep 15;18(9):e0289492. doi: 10.1371/journal.pone.0289492 (PMC10503701; doi:10.1371/journal.pone.0289492)
Supplement: Attachment — Submitted filename: Cabral et al._R1 Plos One_Response to reviewers.docx [file pone.0289492.s002.docx]

**Response to Reviewers**

***Dear reviewer #1,***

***Thank you very much for your time and consideration of our manuscript. Below you will find our responses to your comments. All changes are highlighted in yellow in the manuscript.***

The authors have performed an in vitro study to evaluate the activity of photodynamic therapy (PDT) using the wild-type and a lab-induced miltefosine-resistant strain. The work is interesting and extensive; however, this in vitro experimental study has been done on the non-clinical stage (promastigotes) which is in the gut of the female sandflies and also the culture media. The story can be different when dealing with the clinical stage (amastigotes or Leishman bodies) which is resided inside the macrophage of humans and animal hosts. These two stages are significantly different in terms of physiology and also molecular makeup. The study needs clarification and modifications:

***R. The reviewer is right and the two forms differ physiologically and molecularly. However, the main purpose of this work was to demonstrate the possible mechanisms of action of PDT using DMMB as a photosensitizer focusing on the changes in the lipid metabolism of Leishmania amazonensis, including a miltefosine-resistant strain. As far as we know, there are no reports about the lipidomic analysis of L. amazonensis miltefosine-resistant strains. Besides, this is the first attempt to understand the lipidomics of Leishmania parasites after PDT. We believe this advance will be of interest to a wide range of researchers working on parasitic diseases, including those interested in PDT. However, we understand that further studies are necessary and addressed this issue in the manuscript.***

- The light source should be specified.

***R. We apologize for that. We used a red LED (660 nm peak wavelength, 25 nm full width at half maximum). We included the information in the text.***

- The distance between the light source and the target cells in PDT should be clarified.

***R. The light source illuminates the wells from underneath, with a distance of 4 mm from the bottom of the well. We included this information in the text.***

- The time length used in PDT should be mentioned.

***R. Parasites were irradiated using a red LED at an irradiance of 20 mW/cm^2^ for 2500 s to deliver a dose of 50 J/cm^2^. We included the information in the text.***

- Specify the exact amount of ROS production in PDT.

***R. ROS production was measured by fluorescence using the indicator 2'-7'-dichlorodihydrofluorescein diacetate (DCFH-DA), a dye whose fluorescence is proportionally related to ROS production in live cells. We found that after DMMB-PDT the WT strain produced an average fluorescence intensity of 38410±4200 a.u., while the resistant strain (MFR) produced an average of 50489±10856 a.u., i.e., the MFR strain generates about 24% more ROS than WT.***

- Present fluorescent peaks and their intensity using BMMB.

***R. We added a figure to show the spectra of DMMB.***

***Dear reviewer #2,***

***Thank you very much for your time and consideration of our manuscript. Below you will find our responses to your comments. All changes are highlighted in yellow in the manuscript.***

1. The manuscript is well presented but there are major concerns that should be addressed by the authors. The title should be clear about the in vitro approach of the study, and there are many questions to be answered before to define the PDT-DMMB as a promising strategy of cutaneous leishmaniasis treatment. I highly suggest the authors to modify the title to make it in line with the results and conclusions presented.

***R.*** ***The reviewer is right and we thank your suggestions. We changed the title of the study to “New insights in photodynamic inactivation of Leishmania amazonensis: a focus on lipidomics and resistance" and addressed this point in the text to make clear the aim of the paper.***

2. There was no study of cytotoxicity in mammal cells. As the PDT-DMMB induces ROS production in a nonspecific way, the evaluation of the treatment in host cells is crucial to ensure the safety of the treatment.

***R.*** ***Indeed, the cytotoxic effects of PDT mediated by DMMB have been addressed in our previous studies (Cabral, F. V. Towards new therapeutic strategies for cutaneous leishmaniasis. text, University of Sao Paulo, 2021.*** [***https://www.teses.usp.br/teses/disponiveis/85/85134/tde-18112021-095721/pt-br.php***](https://www.teses.usp.br/teses/disponiveis/85/85134/tde-18112021-095721/pt-br.php)***). We found that PDT was not toxic to fibroblasts or macrophages even at higher light doses or DMMB concentrations. To clarify that, we included this reference in the text.***

3. The study was carry out in promastigote forms only. An efficacy study should also be performed against intracellular amastigote forms, since this is the form present in the cells of the hosts that will be submitted to the treatment.

***R.*** ***Indeed, the potential of DMMB-PDT on intracellular amastigotes has also been demonstrated in our previous studies (Cabral, F. V. Towards new therapeutic strategies for cutaneous leishmaniasis. text, University of Sao Paulo, 2021.*** [***https://www.teses.usp.br/teses/disponiveis/85/85134/tde-18112021-095721/pt-br.php***](https://www.teses.usp.br/teses/disponiveis/85/85134/tde-18112021-095721/pt-br.php)***). However, the present work aimed to demonstrate new insights into the possible PDT mechanisms and a comprehensive study on lipidomics of a WT and a miltefosine-resistant strain of Leishmania parasites before and after PDT. We understand that further studies are necessary, however, we believe our data open an avenue to help combat drug resistance in cutaneous leishmaniasis. To clarify that, we addressed this issue in the text.***

Find below other points that should be addressed by the authors.

• Please keep the results description objective, and avoid the use of subjective sentence as “very”.

***R. Thank you for your suggestion. We carefully revised the description of the results.***

• In the last sentence of abstract, please revise the use of the word “treat”, since the goal is not to treat but to kill Leishmania parasite.

***R. We have changed the word “treat” to “kill” as suggested.***

• Please, give details about the light source, distance from the light source to the bottom plate. Was the irradiation direct or on the plate cover?

***R. We apologize for that. We used a red LED (660 nm peak wavelength, 25 nm full width at half maximum). The experimental setup illuminates the wells from underneath, with a distance of 4 mm from the bottom of the well. We included the information in the text.***

• Was the dilution factor of miltefosine and DMMB 1:2?

***R. Yes, the dilution factor for both compounds was 1:2. We added this information to the manuscript.***

• Why did immunofluorescence microscopy use 8 J/cm2 while the other assays used 50 J/cm2?

***R. Indeed, we used immunofluorescence to understand the role of PDT on parasites. In this case, reducing the light dose to a sublethal dose is more appropriate. We clarified this issue in the manuscript.***

• In lipid analysis, please detail DMMB-PDT treatment.

***R. We added information about DMMB-PDT in the text as per your request.***

• In Figure 1B, was the EC50 value lower than the low DMMB concentration evaluated? If yes, what was the R2 value for this? Would it appropriate to adequate evaluate lower concentrations?

***R. The R^2^ found for DMMB-PDT dose-response curve was 0.98 for both WT and MFR. We added this information to the manuscript. Indeed, we obtained the EC50 values to demonstrate the resistance to miltefosine and the susceptibility of MFR strains to PDT. To conduct the experiments, we used the conditions established in our previous manuscript (Cabral, F. V. et al. Organic Light‐Emitting Diodes as an Innovative Approach for Treating Cutaneous Leishmaniasis. Adv Mat Technol 2021, 2100395).***

• The figure 1 legend missed information, such as miltefosine treatment concentration in fig 1B, DMMB in fig 1A. please revise the legends and add these and other important information.

***R. We added the information about miltefosine treatment in the figure 1.***

• In figure 2, the x-axis title for the different treatment is confuse, please revise. Revise the legend as well.

***R. We revised the x-axis title and legend.***

• It is confuse to follow the different groups of treatment since there is no consistent in the group’s name. Figure 3 caption and legends in the image is very confuse, per example. The authors should be consistent in the denomination of the groups and in the use of terms such as DMMB-PDT throughout the manuscript.

***R. We revised carefully the text to maintain coherence.***

• Please review and alter legend and caption of figure 3.

***R. We revised and changed the legend.***

• The results of mitochondria membrane potential and lipid droplets are presented in the Figures 3 and 4. Can the authors to quantify the fluorescence area in these and other images and present qualitative data that should be statistical evaluated?

***R. Indeed, statistical analysis for these assays would not add new information. Our purpose was to demonstrate the impact of PDT on both Leishmania strains compared to untreated controls. The images clearly show that no differences are noticed comparing both strains.***

• The least sentence of the second paragraph of discussion it is confuse and should be reviewed.

***R. We revised the sentence as per your request.***

• The lack of number line make it difficult to reference text during revision.

***R. We added line numbers in the text.***
