# Supplementary material for: New insights in photodynamic inactivation of Leishmania amazonensis: A focus on lipidomics and resistance
Source: PLoS One. 2023 Sep 15;18(9):e0289492. doi: 10.1371/journal.pone.0289492 (PMC10503701; doi:10.1371/journal.pone.0289492)
Supplement: Attachment — Submitted filename: Cabral et al._R2 Plos One_Response to reviewers.docx [file pone.0289492.s003.docx]

**Response to Reviewers**

***Dear reviewer #1,***

***Thank you very much for your time and consideration of our manuscript. Below you will find our responses to your comments. All changes are highlighted in yellow in the manuscript.***

1 - Do the author mean by "overcome drug resistance in cutaneous leishmaniasis" the use of photodynamic therapy as an adjunct to drug therapy, or is it suggested as a separate solution without the presence of drugs?

***R. Photodynamic therapy (PDT) is an attractive light-based technology that involves the use of a photosensitizer and light to generate reactive oxygen species that can kill microorganisms. Because of its multi-target features, PDT can attack simultaneously lipids, proteins, and nucleic acids. Because of this characteristic PDT is unlikely to produce resistance, as opposed to the current drugs available to treat Leishmaniasis. In addition, PDT has been shown to promote antimicrobial activity against several pathogens, including multidrug-resistant bacteria and some yeast species. However, the use of PDT on drug-resistant Leishmania remains unexplored. We intended to show that PDT can effectively kill both the wild-type and a drug-resistant strain, i.e. miltefosine-resistant. Thus, we propose that PDT can overcome the growing drug resistance problem in cutaneous leishmaniasis by using topically the PS and delivering light directly to the target. We addressed this issue in the manuscript.***

2 - What does it mean by "they absorb light in the red region of the spectrum, and red light penetrates deeper into tissue than shorter wavelengths"? Are the wounds caused by L. amazonensis not on the surface of the skin and is there a need for the presence of light in deeper tissues?

***R. Indeed, light is absorbed by the photosensitizer. However, the red light that is not absorbed may be scattered forward and penetrate deeper into biological tissues. We rephrased the sentence for better/clearer understanding.***

3- It is suggested to measure and investigate the amount of reactive singlet oxygen (1O2) created by the presence of light-sensitive material. In this case, you can refer to the article with doi: 10.1016/j.bpj.2011.12.043.

***R. Indeed, our purpose was not to correlate singlet oxygen generation with PDT dosimetry as Jarvi and collaborators. Here, we intended to verify if resistant Leishmania parasites were susceptible to high levels of ROS generated inside the cells, that they were unable to deal with, subsequently causing cell death***

4- More details of photodynamic therapy conditions need to be explained.

***R. We have added a lot more detail, including light doses and exposure times to the section “Light source and photosensitizer”.***

5- In the discussion of the text of the article, the presence of ROS in light-sensitive species is described, but no information is given about the calculation of these species separately.
***R. The calculation of ROS was given by the values obtained using the indicator DCFH, which is directly proportional to the total ROS production. However, the DCFH indicator can only measure the total amounts of ROS produced by PDT. Thus, we cannot address which ROS species are being generated at that time point evaluated. As above mentioned, our purpose was only to verify if resistant Leishmania parasites were equally susceptible to oxidative stress as the wild-type. Curiously, resistant cells were more susceptible to ROS than wild type. This result indicates that resistant strains were not able to reduce oxidative stress. Indeed, differences in phosphatidylethanolamine content between strains could explain these data. We addressed this issue in the manuscript.***

6- The author should write this work has been done using non-clinical promastigote (an extracellular stage in sandflies and culture media). The story could be different using the intramacrophage amastigote assay before conducting the study in advanced in vivo and clinical models.

***R. We added “Leishmania promastigotes” to the keywords to clarify this issue. However, the potential of DMMB-PDT on intracellular amastigotes has also been demonstrated in our previous studies (Cabral, F. V. Towards new therapeutic strategies for cutaneous leishmaniasis. text, University of Sao Paulo, 2021.*** [***https://www.teses.usp.br/teses/disponiveis/85/85134/tde-18112021-095721/pt-br.php***](https://www.teses.usp.br/teses/disponiveis/85/85134/tde-18112021-095721/pt-br.php)***). In addition, the mechanisms and a comprehensive study on lipidomics of a WT and a miltefosine-resistant strain of Leishmania parasites before and after PDT have still not been reported and this is one of the purposes of this manuscript. We understand that further studies are necessary, but we believe our data here opens novel avenues to help combat drug resistance in cutaneous leishmaniasis and goes someway to an initial understanding of how/why PDT is of therapeutic value against Leishmania.***

***Dear reviewer #2,***

***Thank you again for your time and consideration of our manuscript. Below you will find our responses to your comment. The change is highlighted in yellow in the manuscript.***

In the second version of the manuscript, the authors addressed all my concerns. However, although the new title seems more appropriate now, the authors still need to clarify the in vitro approach of the study in the title of the manuscript.

***R.. We understand that the title and abstract should highlight the scope of the paper and the abstract clearly mentions promastigotes and set as an in vitro study. Thus we have added Leishmania promastigotes to the keywords.***
